# Ranking of Iranian provinces based on healthcare infrastructures: before and after implementation of Health Transformation Plan

**DOI:** 10.1186/s12962-020-0204-5

**Published:** 2020-02-03

**Authors:** Payam Shojaei, Najmeh bordbar, Arash Ghanbarzadegan, Maryam Najibi, Peivand Bastani

**Affiliations:** 1 Department of Management, Shiraz University, Shiraz, Iran; 2 Student Research Committee, Shiraz University of Medical Sciences, Shiraz, Iran; 3 Australian Research Centre for Population Oral Health, Adelaide Dental School, University of Adelaide, Adelaide, South Australia Australia; 4 Health Human Resources Research Center, School of Management and Medical Informatics, Shiraz University of Medical Sciences, Shiraz, Iran

**Keywords:** Health Transformation Plan, Iran, VIKOR method, Shannon’s entropy method

## Abstract

**Background:**

Health Transformation Plan (HTP) was occurred in 2014 to improve access and equity and reduce out of pocket payments in Iranian Health Care System. In this regard the aim of this study is evaluating and ranking the service provider’s infrastructures among the country provinces as an indicator of equity before and after implementation of the HTP.

**Methods:**

This cross sectional study is conducted in 2017. The study population included 31 provinces of the country. Data related to 4 years from 2012 to 2016 were included from the data bases of Ministry of Health and Medical Education as well as the statistics yearbook of the country. The obtained results of multi-criteria decision-making methods were analyzed as well. SPSS_18_ and Excel_2013_ software were used for data analysis.

**Results:**

Based on the VIKOR method, in 2012, Mazandaran, Tehran and Fars provinces and in 2013, the provinces of Tehran, Fars and Isfahan ranked from first to third respectively. Similarly after HTP, in 2015, the provinces of Tehran, Khorasan Razavi and Fars and in 2016 the provinces of Tehran, Fars and Khorasan Razavi have ranked from first to third respectively. Paramedic, dentist, pharmacist, medical institutions and hospital bed had a significant difference before and after the implementation of Health Transformation Plan, so that the number of these indicators increased after implementation of the HTP (*P* value < 0.05).

**Conclusions:**

According to the results, there are many differences between the provinces and these disparities have not decreased significantly after HTP. Consequently, it is suggested to the health sector policy makers to make regional plans and allocate the budget of HTP, based on the status of the provinces. In addition, responding to these inequalities requires a transparent and systematic approach to provide the budget for allocating to the population, health needs, and the lack of development and geographical isolation of regions.

## Background

Fair distribution of financial resources in the health care system helps to reduced health inequalities and health outcomes [1, 2]. Moreover, the right of people to access health services and fair health care has been considered as a major issue by many national and international organizations [3]. Besides, the World Health Organization considers the improvement in health equity both in international and national, as one of the greatest challenges of the new century [4]. On the other hand, various factors of health systems affect the health promotion and reduction of health inequalities, including leadership and stewardship, strategies and policies, health system structure, inter-sectors cooperation, health sector reforms and the mechanisms of allocating health care resources [5]. In this regard, resource allocation as well as healthcare Infrastructures is considered as one of the most important tools for promoting equity in health [6].

Against the emphasis on fair allocation of the resources, the results of various studies indicate the unequal distribution of rural health houses in the provinces of Iran [7], unequal distribution of primary care physicians in different regions of Greece and Albania [8], and unequal distribution of physicians in rural areas of the United States in 2005 [9]. Australia is also the third largest country in terms of regional disputes [10] and increasing the inequality regional disparities is as a serious concern in Russia [11]. While health systems are looking for better ways to meet current and future challenges, and developing countries, including Iran, aren’t exception in this respect.

The budget allocated to health in Iran by 2014 has been less than 8% of the total state budget [12]. In recent years, Iran’s health system has suffered from problems such as the high direct out of pocket payments (OOP) and the increase in households’ catastrophic costs [13]. So that OOP increased from 40.63 to 54.64 percent between 2008 and 2014 [13] as well as the percentage of households faced with catastrophic costs varied from 8.3 to 22.2% by 2012 in different regions of the country [14]. At the same time, the outbreak of chronic diseases in Iran and the accumulation of health centers in large cities have resulted in poor accessibility or even lack of access to services in other deprived areas [15].

Over the past three decades, the Iranian government has made significant efforts to promote health and reduce health inequities through the establishment of a primary health care network (PHC), the implementation of the rural family physician program, rural health insurance program and national health insurance, but there is still a concern about the fair access to health cares [16]. These deficiencies led the policy makers to adopt a reform known as the Health Transformation Plan (HTP). The program follows several goals in a step-by-step process to achieve universal health coverage. One of the most important goals of this program was to reduce the Out of Pocket (OOP), which is considered as the most inefficient and descending financing mechanism [14].

Another important goal of this plan is to prevent referral of patients to centers outside the hospitals affiliated with the Ministry of Health for the purchase of medicines, laboratory and radiological equipment and services, strengthening special clinics and promotion of outpatient care services, supporting the retention of physicians in deprived areas, increasing the presence of specialized physicians residing in hospitals affiliated to the Ministry of Health and improving the hotel accommodation quality in governmental hospitals [17]. Given that the difference in health care services in the various areas of Iran is high and most of the specialized physicians, hospitals, laboratories, medical rehabilitation centers are in the big cities and provincial centers [18], so in this plan, the state has tried to provide the deprived with access to health services and focus on improving the quality of health services provided in deprived areas, and hopes that the implementation of this plan will lead to fair access to health services [19].

The first phase of this HTP, focusing on health services and governmental hospitals affiliated with the Health Ministry, was launched in May 2014; and continued with the modification and updating of medical tariffs on October 2014 [20].

Given that health system, like any other system, is a set of interrelated parts that should work together for effective function. Health infrastructure is one of the components of effective health services provision. Health infrastructure includes buildings, facilities, equipment, personnel and technology, and WHO also considers the effectiveness of infrastructure distribution as an important challenge [5]. There were significant resources in the Health Transformation Plan in Iran, and personnel and facilities increased in the country, but only the number of health care resources is not effective on people’s health, but also the distribution of resources is important. And the unequal distribution of health services is a major obstacle to improve health services provision in world health systems [7].

Regarding what was discussed, it seems that unbalanced distribution of healthcare resources is associated with different providing opportunities and capabilities for the provinces of the country. Considering the most important goal of the Health Transformation Plan means reducing health inequalities in the country, the matter will be more important. Meanwhile, one of the necessary information bases for proper national and regional planning is awareness of the capabilities of different provinces. Therefore, determining the position and status of different provinces in terms of development level before and after implementation of the HTP is very important. In addition, this study was conducted with the aim of evaluating and ranking the provinces of the country based on the healthcare infrastructures before and after implementation of the Health Transformation Plan.

## Methods

**Fig. 1:**
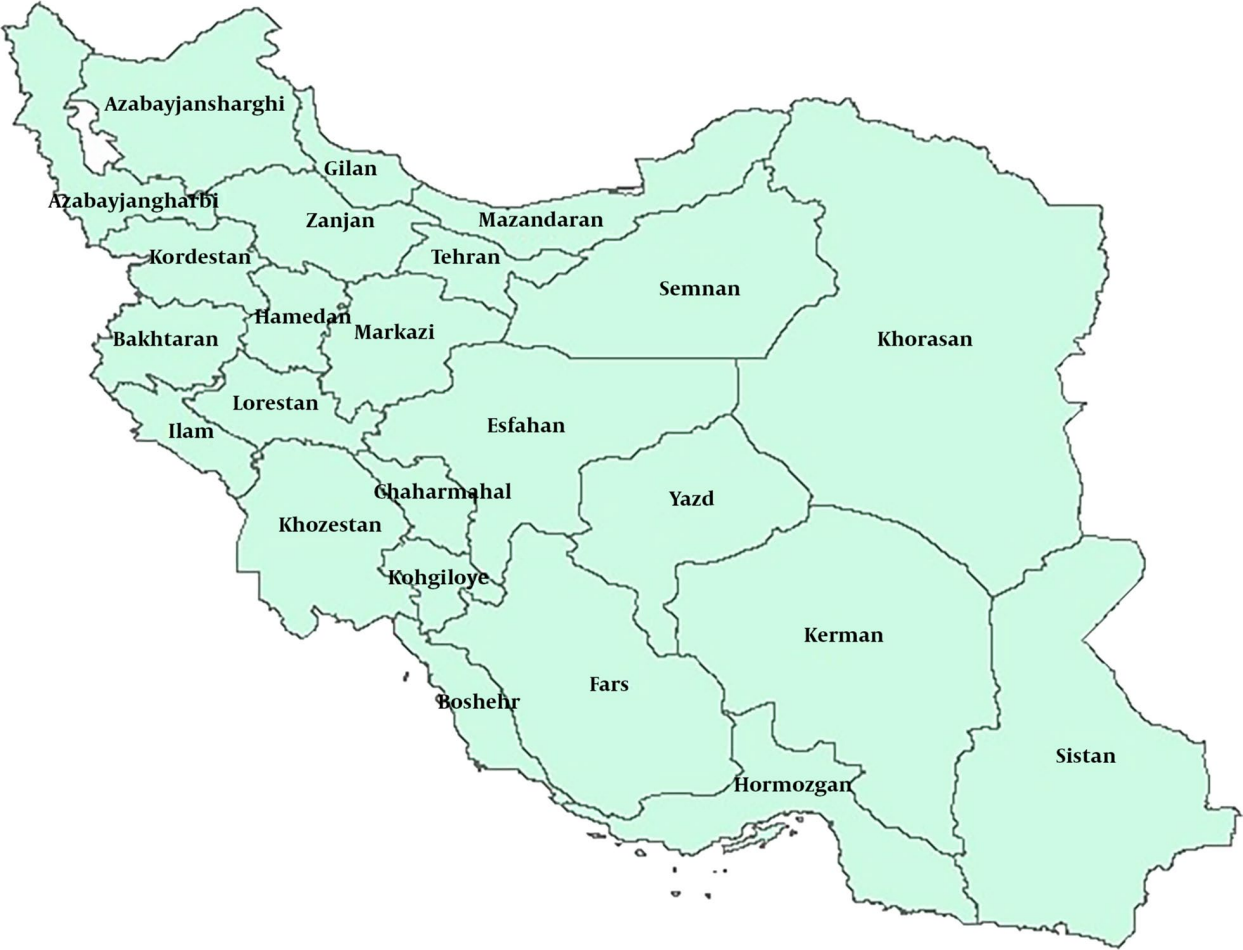
A map of Iranian provinces [21]

This is a cross sectional descriptive-analytic study, conducted in 2017. The study area included 31 provinces of the country (Fig. 1). In this study, the data related to 4 years of the provinces of the country (2012 and 2013 before the development of the HTP), 2015 and 2016 (after the development of the Health Transformation Plan) were studied; and the required statistics and information from the data bases of Ministry of Health and Medical Education as well as statistics yearbook of the country were prepared. Considering that 2014 is the year of implementation of the plan, so the data of 2014 have not been included in the study analysis. On the other hand, based on the four studied years (2 years before implementing HTP and 2 years after that), in addition to perform the ranking, it was possible to examine the process of changes for each indicator in each province. In this regard, multi-criteria decision-making method was applied. This method is one of the decision-making methods in which the problem has multiple criteria and the purpose of the decision is based on these multiple criteria [21]. SPSS_18_ and Excel_2013_ software were used to analyze the data.

In the ranking of the provinces of the country, 11 effective indicators in the field of health and treatment (including paramedics, staffs, general practitioner, dentist, pharmacist, specialists, medical institutions, hospital beds, diagnostic laboratories, rehabilitation centers and pharmacies affiliated with the Ministry of Health, and Medical Education) whose data are available, were used. The selection of such indicators has been based on the objectives of the Health Transformation Plan and having access to available data. In addition, Shannon’s entropy method was used to calculate weights of each of the examined indicators in this study.

The “entropy” is a very important concept in the social sciences, physics and information theory, and when the data of a decision matrix is fully characterized, this method can be used to evaluate weights [22]. In this way, if $m$ is the number of options and $n$ is the number of indicators, the weight of the indicators is obtained briefly by taking the following steps [23]:

- Step 1: Calculating the probability distribution through the following relationship:
  1 $$P_{\,i\,j} = \frac{{a_{\,i\,j} }}{{\sum\limits_{i = 1}^{m} {a_{\,i\,j} } }}$$
- Step 2: Calculating the amount of entropy in which $k = \frac{1}{Lnm}$
  2 $$E_{\,j} = - k\sum\limits_{i = 1}^{m} {P_{\,i\,j} Ln(P_{\,i\,j} )}$$
- Step 3: The amount of uncertainty is obtained.
  3 $$d_{j} = 1 - E_{j}$$
- Step 4: Calculating the weights of the indicators will be calculated by the following equation.
  4 $$w_{j} = \frac{{d_{j} }}{{\sum\limits_{i = 1}^{n} {d_{j} } }}$$

The VIKOR method was used to rank the provinces of the country. The VIKOR method is one of the multi-criteria decision-making methods introduced by Opriukovich and Tsugon in 1998.

This method evaluates issues with inappropriate and incompatible criteria. It has been developed to multi criteria optimization of the developed complex systems; and is as a determiner of a list of compromise ratings, compromise and intervals solutions for fixing the weight of the criteria. This method focuses on ranking and selection of alternatives despite existing conflicting criteria.

VIKOR introduces a multi-criteria ranking index based on a specific criteria of proximity to the ideal solution. In this method, it is assumed that each alternative is evaluated according to the criteria function, and compromise ranking can be made by comparing the criteria of proximity with the ideal alternative.

An aggregate LP metric function is used for a compromise rating [24].

Suppose that the $J$ alternative is specified with $a_{1} \,,\,a_{2} \,,\, \ldots ,\,a_{j}$. For $a_{j}$ alternative, the degree and the amount of degree of $j$ is determined by $f_{ij}$. That is, $f_{ij}$ is the value of the criterion function of $i$ for the $a_{j}$ alternative, in a way that, $n$ is the number of criteria. The development of the VIKOR’s method begins with the following form called the LP metric:

5 $$\begin{aligned} L_{P,j} = \left\{ {\left. {\sum\limits_{i = 1}^{n} {\left[ {w_{i} \,(f_{i}^{*} - f_{ij} )/(f_{i}^{*} - f_{i}^{ - } )} \right]^{P} } } \right\}} \right.^{{\frac{1}{P}}} \, \hfill \\ \,\,\,\,\,\,\,\,\,\,\,\,\,\,\,\,1 \le P \le \infty \,\,\,\,\,\,\,\,\,\,j = 1\,,\,2\,,\, \ldots \,,\,J\,\,\,\, \hfill \\ \, \hfill \\ \end{aligned}$$

Based on the VIKOR method, the value of $L_{1,j}$ (as $S_{j}$ in equation (1) and $L_{\infty ,j}$ [as $R_{j}$ in equation (2) is used to formulate the ranking criterion.

The obtained result from $\hbox{min} \,S_{j}$ expresses the maximizing group desirability (the rule of the majority) and the obtained results of $\hbox{min} \,R_{j}$, expresses minimizing the regret of individuals from the opponent alternatives.

**Fig. 2:**
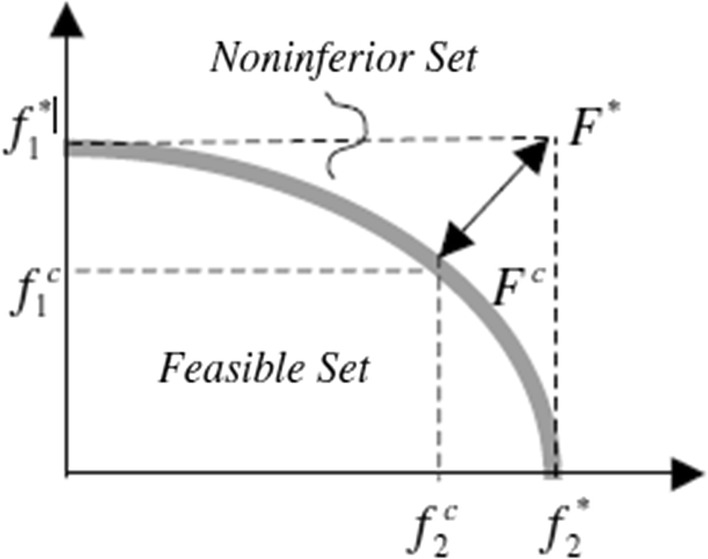
Ideal and compromise solutions

An agreement solution of $F^{c}$ is a justifiable answer to an ideal solution of $F^{*}$, and compromise means an agreement reached through bilateral negotiations as depicted in Fig. 2:

6 $$\Delta f_{1} = f_{1}^{*} - f_{1}^{c} \,\,\,\,\,\,\,\,\Delta f_{2} = f_{2}^{*} - f_{2}^{c} \,$$

The VIKOR’s compromise ranking algorithm includes the following steps: [24]

- Step 1: Determining the best and worst value of $f_{i}^{ - }$ for all criteria functions $(i = 1\,,\,2\,,\, \ldots ,\,n\,)$ if $i$ th function represents an advantage (positive aspect) in this case $f_{i}^{*} = \mathop {\hbox{max} }\limits_{j} \,f_{ij}$ and $f_{i}^{ - } = \mathop {\hbox{min} }\limits_{j} \,f_{ij}$.
- Step 2: Calculating the values $S_{j}$ and $R_{j}$ for $j = 1\,,\,2\,,\, \ldots ,\,J$ using the following relationships:
  7 $$P = 1\,\,\, \Rightarrow \,\,S_{j} = \sum\limits_{i = 1}^{n} {w_{i} } \,\frac{{f_{i}^{*} - f_{ij} }}{{f_{i}^{*} - f_{i}^{ - } }}$$
  8 $$P \to \infty \,\,\, \Rightarrow \,\,R_{j} = \hbox{max} \,\left[ {w_{i} \,\frac{{f_{i}^{*} - f_{ij} }}{{f_{i}^{*} - f_{i}^{ - } }}\,} \right]$$
  In a way that $w_{i}$ expresses the relative weight of each criteria and indicates the relative importance of each one.
- Step 3: Calculating the amount of $Q_{j}$ for $j = 1\,,\,2\,,\, \ldots \,,\,J$ using the following equation:
  9 $$Q_{j} = \,v\,\left( {\frac{{S_{j} - S^{*} }}{{S^{ - } - S^{*} }}} \right) + (1 - v)\,\left( {\frac{{R_{j} - R^{*} }}{{R^{ - } - R^{*} }}} \right)$$
  In a way that:
  $$S^{*} = \hbox{min} \,S_{j}, \quad S^{ - } = \hbox{max} \,S_{j}, \quad R^{*} = \hbox{min} R_{j}, \quad R^{*} = \hbox{max} R_{j} \,$$
  In this regard, the weight of the strategy is introduced “the majority of the criteria” (or the maximum utility of the group), which is here $v = 0.5$.
- Step 4: Ranking alternatives by sorting descending values of $Q\,,\,R\,,\,S$. So the results of the three lists are ranked.
- Step 5: It is suggested that a compromise solution to be considered for the alternative $(a^{\prime})$ ranking by the minimum criteria of $Q$ if two conditions are met.

Condition 1. Acceptable advantage: it should be $Q(a^{\prime\prime}) - Q(a^{\prime}) \ge \,DQ$. In a way that $(a^{\prime\prime})$ is an option having the second position in the classified list of $Q$ and $DQ = \frac{1}{J - 1}$ so that $J$ is the number of alternatives.

Condition 2. Acceptable stability in decision making: Alternatives $(a^{\prime})$ should have the best rank in the list $\,R\,,\,S$. That is, this reconciliation must be stable in a decision making process. So that under any circumstances (majority voting $v > 0.5$, agreement with $v = 0.5$, veto with $v < 0.5$) to be set.

If one of the conditions is not met, a set of compromise solutions is suggested:

A. Options $(a^{\prime})$ and $(a^{\prime\prime})$ if the only second condition is not meet.
B. Options $(a^{\prime})$, $(a^{\prime\prime}), \ldots ,(a^{M} )$ if the first condition does not occur and $(a^{M} )$ is determined from the following equation: $Q(a^{M} ) - Q(a^{\prime}) < \,DQ$ M for maximum.

The best alternative ranked by the $Q$ index is the amount with the minimum $Q$ quality. The method of VIKOR is a useful tool in multi-criteria decision making, especially when the decision maker is not able to express its preferences at the beginning of the system design. In order to compare the data before and after the Health Transformation Plan, after examining the normality of the data, the paired t-test was used.

## Results

In order to prioritize the alternatives, it is necessary to extract the weight of the criteria at first. As previously mentioned, using the Shannon entropy method and applying the formulas to, the weight of the criteria was extracted each year, and the final weight was obtained based on the average in Table 1 (1, 4). Since the number of alternatives for prioritizing is equal to 31, in formula, we put m = 31 for computation k in order to calculate the value of entropy (2). Therefore, the k value is 0.291, which remains constant during the calculation. According to the entropy method, given the specific criteria, the greater deviations result in greater weight. Table 1 shows the results of Shannon’s entropy. According to the table, the number of pharmacies and with small difference, the number of rehabilitation centers indexes had the maximum weight; and the index of paramedic number had the lowest weight among the understudy indicators.

**Table 1:** Weight of the indexes studied by entropy method

Pharmacy	Rehabilitation centers	Diagnostic lab	Hospital bed	Medical institution	Specialist	Pharmacist	Dentist	General medicine	Staffs	Paramedic	Indicators
0.145	0.143	0.066	0.083	0.049	0.108	0.111	0.079	0.067	0.08	0.063	Weights

Having calculated the weight, the decision matrix must be normalized to determine the priority of the alternatives. According to Opricovic and Tzeng, in order to implement VIKOR the use of linear normalization provide satisfactory solutions [24, 25]. This is mainly because of not depending values to evaluation unit of a criterion function. Then, it is necessary to determine best and worst value of all criteria. The best value is the ideal solution so that for the criteria that are inherently positive, the larger the number we are closer to the ideal. On the other hand, if the criterion is intrinsically negative, the lower is the ideal state. The negative ideal solution is in contrast to this. In other words, a negative ideal means having high values for intrinsically negative and small amounts for intrinsically positive indicators. Accordingly, for each decision matrix in each year, positive and negative ideal values were calculated, and the values of S, R, and Q were obtained using formulas (7) to (9). Furthermore, the value of v was considered to be 0.5 ($v = 0.5$) implying the weight of the strategy of ‘the majority of criteria’’ in VIKOR method. The values of Q and R for each of the alternatives (provinces) were reported based on the data of 2012 and 2013 that were before implementing the Health Transformation Plan; and 2015 and 2016 that are after implementing the mentioned plan: If the values of Q and R are set in descending form, two ranked tables will be obtained.

As it is shown in Table 2, based on the VIKOR method, in 2012, Mazandaran, Tehran and Fars provinces ranked from first to third. Since the condition (1) Acceptable advantage is done in the VIKOR method, the acceptance benefit condition is confirmed.

**Table 2:** Ranking of provinces in the country based on Q and R values in 2012 and 2013 (before implementing the Health Transformation Plan)

Province	Q	Ranking of province (in 2012) Based on Q	Province	R	Ranking of province (in 2012) Based on R	Province	Q	Ranking of province (in 2013) Based on Q	Province	R	Ranking of province (in 2013) Based on R
Mazandaran	0.2743	1	Mazandaran	0.1192	1	Tehran	0	1	Tehran	0.0561	1
Tehran	0.3155	2	Tehran	0.176	2	Fars	0.2464	2	Fars	0.0755	2
Fars	0.5187	3	Fars	0.1784	3	Isfahan	0.349	3	Isfahan	0.0802	3
Razavi Khorasan	0.6486	4	Sistan and	0.1875	4	Razavi Khorasan	0.3756	4	Razavi Khorasan	0.0872	4
Isfahan	0.7065	5	Zanjan	0.1911	5	Mazandaran	0.5052	5	Mazandaran	0.0973	5
Khuzestan	0.7225	6	East Azarbaijan	0.1935	6	Khuzestan	0.5777	6	Khuzestan	0.1073	6
Sistan and	0.7595	7	Razavi Khorasan	0.1959	7	Sistan and	0.6635	7	Lorestan	0.1089	7
						Baluchestan					
West Azarbaijan	0.7977	8	West Azarbaijan	0.1965	8	Kerman	0.6696	8	Sistan and	0.109	8
									Baluchestan		
East Azarbaijan	0.8223	9	Khuzestan	0.1965	9	West Azarbaijan	0.6789	9	Gilan	0.1127	9
Kerman	0.8261	10	Golestan	0.1995	10	Gilan	0.692	10	Semnan	0.1141	10
Golestan	0.8571	11	Isfahan	0.202	11	Lorestan	0.7183	11	West Azarbaijan	0.1141	11
Zanjan	0.8746	12	Kerman	0.202	12	East Azarbaijan	0.7402	12	Kerman	0.1174	12
Gilan	0.8931	13	Markazi	0.2044	13	Kermanshah	0.7579	13	Kermanshah	0.1191	13
Lorestan	0.9165	14	Ardebil	0.2062	14	Semnan	0.7937	14	Yazd	0.1208	14
Markazi	0.9181	15	Alborz	0.2068	15	Chaharmahal	0.8114	15	Zanjan	0.1208	15
						and Bakhtiari					
Hamedan	0.9222	15	Hormozgan	0.2068	16	Yazd	0.8127	16	Chaharmahal	0.1241	16
									and Bakhtiari		
Kermanshah	0.9244	17	Eilam	0.2074	17	Golestan	0.8215	17	Golestan	0.1258	17
Hormozgan	0.9327	18	Semnan	0.2086	18	Zanjan	0.83	18	Qom	0.1275	18
Alborz	0.9457	19	South Khorasan	0.2092	19	Hamedan	0.8496	19	Markazi	0.1291	19
Ardebil	0.9509	20	North Khorasan	0.2092	20	Markazi	0.8675	20	South Khorasan	0.1291	20
Yazd	0.9524	21	Qom	0.2092	21	Alborz	0.8865	21	Alborz	0.1291	21
Kordestan	0.9542	22	Kordestan	0.2092	22	Qom	0.895	22	Hamedan	0.1308	22
Semnan	0.9575	23	Qazvin	0.2098	23	South Khorasan	0.9051	23	Qazvin	0.1308	23
Chaharmahal	0.9582	24	Lorestan	0.2098	24	Hormozgan	0.9062	24	Hormozgan	0.1325	24
Qazvin	0.9582	25	Kohgiluyeh and	0.211	25	Bushehr	0.9121	25	Bushehr	0.1325	25
Qom	0.9714	26	Bushehr	0.2116	26	Qazvin	0.918	26	Kordestan	0.1359	26
Eilam	0.9741	27	Chaharmahal	0.2116	27	Kordestan	0.9254	27	North Khorasan	0.1359	27
Bushehr	0.9749	28	Kermanshah	0.2116	28	North Khorasan	0.9439	28	Kohgiluyeh and	0.1375	28
									Boyer-Ahmad		
North Khorasan	0.9751	29	Hamedan	0.2116	29	Ardebil	0.9481	29	Ardebil	0.1375	29
South Khorasan	0.9779	30	Gilan	0.2122	30	Kohgiluyeh and	0.9617	30	East Azarbaijan	0.1375	30
						Boyer-Ahmad					
Kohgiluyeh and	0.9892	31	Yazd	0.2122	31	Eilam	1	31	Eilam	0.1392	31

$$Q(a) - Q(a) = 0.3155 - 0.2743 \ge \frac{1}{31 - 1}$$

In addition, based on the VIKOR method, in 2013, the provinces of Tehran, Fars and Isfahan ranked from first to third respectively. Since the condition (1) Acceptable advantage is done in the VIKOR method, the acceptable benefit condition is confirmed.

$$Q(a) - Q(a) = 0.2464 - 0.000 \ge \frac{1}{31 - 1}$$

As shown in Table 2, since the R ranking is similar to Q, then for the years 2012 and 2013, the second condition, that is, the acceptance of the decision making, will be met.

On the other hand, according to the VIKOR method, in 2015, the provinces of Tehran, Khorasan Razavi and Fars gained the rank of first to third respectively. Since the condition (1) Acceptable advantage is done in the VIKOR method, the acceptable advantage condition is approved.

$$Q(a) - Q(a) = 0.444 - 0.000 \ge \frac{1}{31 - 1}$$

Based on the VIKOR method, in 2016 the provinces of Tehran, Fars and Khorasan Razavi have ranked from first to third respectively. Since the condition (1) Acceptable advantage is done in the VIKOR method, the acceptable advantage condition is confirmed.

$$Q(a) - Q(a) = 0.4157 - 0.00 \ge \frac{1}{31 - 1}$$

As shown in Table 3, since the R ranking is similar to Q, then for the years 2015 and 2016, the second condition, that is, the acceptance of the decision making, will be met.

**Table 3:** Ranking of provinces in the country based on Q and R values in 2015 and 2016 (after implementing the Health Transformation Plan)

Province	Q	Ranking of province (in 2015) Based on Q	Province	R	Ranking of province (in 2015) Based on R	Province	Q	Ranking of province (in 2016) Based on Q	Province	R	Ranking of province (in 2016) Based on R
Tehran	0	1	Tehran	0.0676	1	Tehran	0	1	Tehran	0.0485	1
Razavi Khorasan	0.444	2	Razavi Khorasan	0.139	2	Fars	0.4157	2	Razavi Khorasan	0.0988	2
Fars	0.5124	3	Isfahan	0.1414	3	Razavi Khorasan	0.436	3	Fars	0.1	3
Isfahan	0.5368	4	Fars	0.1438	4	Isfahan	0.4977	4	Isfahan	0.1024	4
Mazandaran	0.6333	5	Mazandaran	0.1487	5	Mazandaran	0.6364	5	Sistan and	0.1133	5
									Baluchestan		
Sistan and	0.783	6	Sistan and	0.1595	6	Khuzestan	0.6523	6	Yazd	0.1169	6
Baluchestan			Baluchestan								
Kerman	0.8062	7	Semnan	0.1668	7	Sistan and	0.7228	7	Hamedan	0.1193	7
						Baluchestan					
East Azarbaijan	0.8074	8	Lorestan	0.168	8	Kerman	0.731	8	Mazandaran	0.1193	8
West Azarbaijan	0.8098	9	Khuzestan	0.1716	9	East Azarbaijan	0.7342	9	Khuzestan	0.1205	9
Gilan	0.8412	10	Kermanshah	0.174	10	Yazd	0.7715	10	Semnan	0.1253	10
Kermanshah	0.8471	11	Kerman	0.174	11	Hamedan	0.7768	11	Kerman	0.1265	11
Lorestan	0.8483	12	Zanjan	0.1753	12	Kermanshah	0.8007	12	Kermanshah	0.1277	12
Khuzestan	0.8587	13	West Azarbaijan	0.1753	13	West Azarbaijan	0.8106	13	Lorestan	0.1338	13
Semnan	0.872	14	Markazi	0.1777	14	Gilan	0.8243	14	Markazi	0.135	14
Markazi	0.8829	15	Yazd	0.1789	15	Semnan	0.8556	15	Gilan	0.1362	15
Golestan	0.8857	16	Gilan	0.1789	16	Markazi	0.8642	16	Qazvin	0.1362	16
Hamedan	0.8932	17	Golestan	0.1789	17	Lorestan	0.8723	17	Alborz	0.1362	17
Yazd	0.8999	18	Hamedan	0.1801	18	Alborz	0.8763	18	Zanjan	0.1374	18
Zanjan	0.902	19	Qom	0.1801	19	Golestan	0.8864	19	West Azarbaijan	0.1374	19
Hormozgan	0.903	20	Alborz	0.1801	20	Hormozgan	0.8945	20	East Azarbaijan	0.1374	20
Alborz	0.9212	21	Hormozgan	0.1813	21	Qazvin	0.9019	21	Ardebil	0.1386	21
Chaharmahal	0.9289	22	Qazvin	0.1813	22	Eilam	0.9196	22	Hormozgan	0.1398	22
and Bakhtiari											
Eilam	0.9378	23	Chaharmahal	0.1813	23	Zanjan	0.9215	23	Qom	0.1398	23
			and Bakhtiari								
Qazvin	0.9403	24	South Khorasan	0.1825	24	Chaharmahal	0.9254	24	Golestan	0.141	24
						and Bakhtiari					
Qom	0.9454	25	Bushehr	0.1849	25	Ardebil	0.9271	25	Chaharmahal	0.141	25
									and Bakhtiari		
South Khorasan	0.9485	26	Kordestan	0.1861	26	Bushehr	0.9464	26	South Khorasan	0.1422	26
Bushehr	0.9503	27	North Khorasan	0.1861	27	Qom	0.9465	27	Bushehr	0.1446	27
Kordestan	0.9548	28	East Azarbaijan	0.1861	28	South Khorasan	0.948	28	Kohgiluyeh and	0.1458	28
									Boyer-Ahmad		
North Khorasan	0.968	29	Ardebil	0.1873	29	Kordestan	0.9523	29	Kordestan	0.1458	29
Ardebil	0.9688	30	Kohgiluyeh and	0.1886	30	North Khorasan	0.9814	30	Eilam	0.1458	30
			Boyer-Ahmad								
Kohgiluyeh and	1	31	Eilam	0.1886	31	Kohgiluyeh and	0.9879	31	North Khorasan	0.1482	31
Boyer-Ahmad						Boyer-Ahmad					

In order to compare the mean of the understudy indicators 2 years before and 2 years after the implementation of Health Transformation Plan, the paired t-test was used. The results of this test are shown in Table 4.

**Table 4:** Comparison of the understudy indicators two years before and two years after implementation of the Health Transformation Plan

No.	Indicators	Mean (2 years before implementation of HTP)	Mean (2 years after implementation of HTP)	P-value
1	Paramedic	6800	8092	0.002
2	Staff	4010	4024	0.959
3	General practitioner	473	503	0.141
4	Dentist	102	127	0.004
5	Pharmacist	48	63	0.001
6	Specialist	534	534	0.996
7	Medical institutions	17	19	0
8	Hospital bed	2995	3391	0.001
9	Diagnostic Lab	70	74	0.126
10	Rehabilitation centers	14	17	0.157
11	pharmacy	22	19	0.627

As the figure shows this country has 31 provinces that all are administrated by the central government.

According to the results of Table 4, paramedic, dentist, pharmacist, medical institutions and hospital bed had a significant difference before and after the implementation of Health Transformation Plan, so that the number of these indicators increased after implementation of the HTP (P-value < 0.05).

## Discussion

Studies in the field of inequality indicate the importance of reallocation of resources in health policies and decisions make on health field, and developing countries including Iran, through general coverage of insurance, rural insurance, and family physicians have been trying to reduce these inequalities [26]. On the other hand, unequal distribution of health services is a major barrier to provision of improving health and treatment in the health systems across the world, and there is a link between access to health care resources and health status [27]. Therefore, understanding the mode of distribution of health facilities among the provinces of the country and analyzing them to identify the deficiencies and provincial failures is considered as the first step in the health and treatment planning. Organizing shortcomings through logical and prioritized planning for the provinces is considered as a second step.

The findings of this study indicate that in 2012 and 2013, as the years before implementation of the Health Transformation Plan, the provinces of Mazandaran, Tehran and Fars in 2012 and Tehran, Fars, Isfahan in 2013 in terms of investigating the developing indicators have been in a better position respectively. These results are probable and inevitable because Tehran is the political capital of the country and most of the health facilities are concentrated in this city. Isfahan, Fars and Mazandaran as three other metropolitans of the country had the same situation. In another words, most of the educated workforce in medical and para medical scopes have tendency to work in these cities with higher living facilities and higher incomes as well. In this regard Health Transformation Plan tries to move toward equality by reallocate the health infrastructures all over the county But as the present results indicated, the status of these indicators in 2015 and 2016 implies that the provinces of Tehran, Khorasan Razavi and Fars in 2015, Tehran, Fars and Khorasan Razavi, are respectively in better position than other provinces of the country. As we emphasized before, it should be noted that these provinces are as the megacities of the country; and the main focus of health and treatment facilities is on these provinces. Therefore, these results are not far-reaching and, given the level of existing facilities in these provinces, the results are logical although they lead to the reality that implementing of HTP can not differ the status of the poor regions from the perspective of health facilities.

The results of Kazemi et al. [28] and Bahrami et al. [18] confirmed the results of this study and reported these provinces as developed and enjoyed ones. On the other hand, in 2012 and 2013, respectively, the provinces of Kohkiluyeh and Boyerahmad, South Khorasan, North Khorasan in 2012 and Eilam, Kohkiluyeh and Boyer Ahmad and Ardebil in 2013 were in the worst situation in terms of enjoying the under study indicators. The stated provinces are all under developed and territory regions with lower level of incomes and social determinant of health status along with less physical facilities and lower tendencies of educated work force for staying there.

As other results show, In 2015 and 2016, as the years after implementation of the Transformation Plan, respectively, the provinces of Kohkiluyeh and Boyerahmad, Ardebil, North Khorasan in 2015, and Kohkiluyeh and Boyerahmad, North Khorasan and Kurdistan, are in the lowest status of enjoying the considered indicators. These provinces are as the deprived and less developed regions of the country; they have remained relatively less enjoyed after the implementation of the Transformation Plan. It well indicates that HTP helps the developed provinces become flourished and more developed and in contrast, the under developed provinces remained deprived and needful.

By investigating 10 provinces with the lowest Q values (best situation) and comparing them in the years before and after the Transformation Plan, the slight changes are seen in these rankings, and most provinces are almost constant at these 10 rankings. On the other hand, investigating the situation of 10 provinces with the highest Q values and in the worst conditions in terms of the understudy indicators that are mostly as deprived and poor provinces, show the similar results with the previous obtained results; and almost ranking the provinces is fixed and without change.

In recent years, little research has been carried out on the performance of the provinces in the infrastructures of health and treatment area in macro scale; and most of the studies have been conducted on the ranking the counties of province regarding enjoy the health facilities level.

Amini et al. [29] ranked the provinces of the country in terms of health; and the results showed that the provinces of Isfahan, Tehran and Markazi were in good health situation. They report that the situation of Khouzestan, Sistan and Baluchestan and Kohkiluyeh and Boyer Ahmad provinces are critical [29].

In the same study in Serbia, there are also severe regional inequalities between the north and the south, urban and rural areas, as well as the central and peripheral regions. In this country, the province of Vojvodina and the city of Belgrade show a greater development than other regions of Serbia, especially in the southeastern regions. These inequalities become more and more day by day [30].

The results of this study indicate that the Health Transformation Plan has not been so successful in reaching its goals. Its goal was the equality in access to health services and justice in distribution of these services. Now, the provinces that were in good condition before the implementation of the plan and are at the central region of the country, after the implementation of the Health Transformation Plan has found a better situation. They are still in a better position. Besides, the provinces that had the least access to health services before the implementation of the Health Transformation Plan, and were generally peripheral and poor provinces, are still in the worst conditions after implementation of the plan.

It seems that there are many differences in terms of distribution of health indicators in the provinces of the country; and the provinces in the border are at the lowest level of enjoying. Furthermore, despite the implementation of the Health Transformation Plan, no significant changes in the status of the provinces were observed in comparison with the before implementing this plan. Based on the findings of a study conducted by Himanshu et al. the first 6 years of the National Rural Health Mission in India has increased the coverage of mothers’ health care and has reduced regional inequality unevenly [31]. The results of the study done by Dai et al. also showed that regional inequalities in financing the new cooperative medical scheme in Jiangsu weakens the equality of using healthcare between the regions; therefore it is necessary that a fairer financing to be existed to reduce regional inequalities [32]. However, Shikeri et al. conducted a study on measuring the health indicators in different regions of Tunisia, and the obtained results show a decreasing trend in regional disparities in recent years, which is inconsistent with the results of the present study [33].

## Conclusions

According to the results of this research, there are many differences between the provinces of the country and these disparities have not decreased significantly after the implementation of the Transformation Plan. Consequently, it is suggested to the planners and officials of the health and treatment sector to make regional plans and allocate the budget of Health Transformation plan, based on the status of the provinces. In addition, responding to these inequalities requires a transparent and systematic approach to providing the budget for allocating to the population, health needs, and the lack of development and geographical isolation of regions.

## Data Availability

All datasets are publicly available cited sources.
